# Research on Distributed Multi-Sensor Cooperative Scheduling Model Based on Partially Observable Markov Decision Process

**DOI:** 10.3390/s22083001

**Published:** 2022-04-14

**Authors:** Zhen Zhang, Jianfeng Wu, Yan Zhao, Ruining Luo

**Affiliations:** Air Defense and Missile Defense College, Air Force Engineering University, Xi’an 710051, China; zz932785956@163.com (Z.Z.); zytyler@163.com (Y.Z.); luo_ruining@163.com (R.L.)

**Keywords:** distributed defense, multi-sensor scheduling, partially observable Markov decision process, intelligent optimization algorithm

## Abstract

In the context of distributed defense, multi-sensor networks are required to be able to carry out reasonable planning and scheduling to achieve the purpose of continuous, accurate and rapid target detection. In this paper, a multi-sensor cooperative scheduling model based on the partially observable Markov decision process is proposed. By studying the partially observable Markov decision process and the posterior Cramer–Rao lower bound, a multi-sensor cooperative scheduling model and optimization objective function were established. The improvement of the particle filter algorithm by the beetle swarm optimization algorithm was studied to improve the tracking accuracy of the particle filter. Finally, the improved elephant herding optimization algorithm was used as the solution algorithm of the scheduling scheme, which further improved the algorithm performance of the solution model. The simulation results showed that the model could solve the distributed multi-sensor cooperative scheduling problem well, had higher solution performance than other algorithms, and met the real-time requirements.

## 1. Introduction

In the context of distributed defense [1], sensors are deployed in a decentralized manner. Multi-sensor collaborative scheduling is a multi-sensor resource management problem. Traditional multi-sensor scheduling research is often aimed at static problems. However, with the development of science and technology, the goal is gradually with the characteristics of high mobility, stealth and changeable tactics, the real battlefield is often complex and changeable, which makes the multi-sensor scheduling process more complicated.

Therefore, how to reasonably schedule multi-sensors in a dynamically changing battlefield and continuously detect and track targets with high precision has become a research hotspot.

In terms of the sensor scheduling model, Vikram et al. for example, used the hidden Markov principle to build a sensor network scheduling model for the target detection problem, and used stochastic dynamic programming to solve the optimal scheduling strategy [2]. Atiyeh et al. used interactive multi-model and particle filter algorithms for maneuvering targets to solve the sensor scheduling selection problem in the target tracking process [3]. Ying He et al. solved the sensor scheduling problem in the target tracking process by using the Monte Carlo sampling method based on the Markov decision principle [4]. Wendong Xiao et al. saved energy consumption on the premise of ensuring target tracking accuracy, and proposed a new adaptive sensor scheduling method [5]. In the process of studying sensor network scheduling, Bo Hu et al. proposed an approximate solution algorithm C-QMDP based on the POMDP model, which reduced the cumulative loss and online computation [6]. Using POMDP and FISST theory, Wei Li et al. proposed a dual-sensor control scheme to maximize the overall utility of the monitoring system [7]. For decentralized large-scale multi-target tracking under the RFS framework, Feng Lian et al. proposed a new sensor selection optimization algorithm based on the marginalized delta-generalized labeled multi-Bernoulli RFS to reduce the computational cost of sensor selection and the accuracy of MTT [8]. Gongguo Xu et al. constructed a sensor scheduling model based on Bayesian theory, and transformed the sensor movement selection problem into a decision tree problem to obtain the optimal sensor combination and effectively solve the sensor scheduling problem [9].

In terms of target tracking, the basic idea is to use the tracking filter algorithm to update the state of the system. At present, the commonly used filtering algorithms mainly include Kalman filter, extended Kalman filter, unscented Kalman filter, particle filter and their improved algorithms. For example, Xiaofei Zhang et al. used the Kalman filter based on the interactive multi-model to process dynamic tracking data, which has a stronger tracking adaptability than a single-model Kalman filter [10]. Bo Lv et al. improved the extended Kalman filter by using innovation theory, an emerging orbit prediction model was built, which effectively reduced the error caused by noise and improved the prediction accuracy of the real-time motion state of the target [11]. In different parts of the unscented Kalman filter algorithm, Bo Chen et al. used statistics and analysis principles to linearize the state estimation, which enhanced the ability to deal with nonlinear problems and improved the tracking effect of the algorithm [12]. Jianyang Hu et al. used the particle swarm optimization algorithm to improve the particle degradation phenomenon, effectively improving the accuracy of target tracking [13].

In terms of resource allocation, with the development of intelligent optimization algorithms, its applications in sensor scheduling problems are becoming more and more extensive. For example, for the sensor scheduling problem of target detection, Lei An et al. compared and analyzed the solution strategies of various intelligent optimization algorithms based on part of the objective Markov decision process, and proved the superiority of the intelligent optimization algorithm [14]. Bans E et al. used a genetic algorithm for a policy search to solve the multi-agent planning problem under the partially observable Markov model [15]. Bo Wang et al. proposed a sensor management method based on real-valued particle swarm optimization algorithm, which could perform sensor-target management more effectively [16]. Ganlin Shan et al. proposed a sensor scheduling method for multi-target detection based on risk theory, and used the improved artificial bee colony algorithm to solve the scheme, which proved the feasibility of the model and the effectiveness of the algorithm [17]. Xiaojuan Zhu et al. proposed an energy-minimizing dynamic task-scheduling algorithm. The improved ant colony algorithm was used to solve the problem, which effectively reduced the task allocation time and energy consumption [18]. For high-threat targets, Yuqi Lan used the binary particle swarm optimization algorithm to solve the multi-sensor multi-target model, which effectively improved the allocation efficiency of sensor resources to targets with different threats [19].

With the development of technologies such as 5G, 6G, and the Internet of Things (IoT), another idea is to improve the sensor communication level based on the existing model. By applying 5G, 6G and other technologies to sensor networks, the intelligence level, collaborative detection accuracy, and anti-jamming capabilities of sensor networks can be improved, and the energy consumption of the network can be reduced to achieve the purpose of optimizing operations. For example, Manzoor Ahmed et al. combined non-orthogonal multiple access (NOMA) with backscatter sensor communication (BSC) and connected them with IoT technology, and proposed a new IoT optimization framework, which optimized the power distribution and effectively improved the network performance under imperfect successive interference cancellation (SIC) [20]. In the case of channel uncertainty, Asim Ihsan et al. proposed a two-stage alternating optimization algorithm to maximize the sensor network energy efficiency (EE) with low complexity by optimizing the transmit power of carrier emitter (CE) and the RCs of RSs [21]. Under the conditions of future high and new technology, these ideas have reference significance for improving the task planning capability of sensor networks under complex conditions.

In distributed cooperative air defense operations, sensors are required to track targets continuously, accurately and quickly, and the scheduling process must be continuous, accurate and fast. Most of the existing research analyzes a certain aspect, which makes the performance of the model and algorithm not comprehensive, and it is difficult to meet the requirements of the battlefield for the model and algorithm, so it is difficult to truly adapt to the real battlefield environment.

Therefore, this paper proposes a distributed multi-sensor cooperative detection model based on the partially observable Markov decision process. The core of this paper is divided into three parts. The first part is the model. Because the target has complex uncertainties such as maneuvering and stealth, the sensors are often incompletely observed; at the same time, in the process of target detection, the scheduling strategy of the sensor at the current moment will affect the observation results of the sensor on the target, the observation results will affect the current estimation of the target state, and the state estimation will affect the scheduling strategy of the sensor at the next moment, which determines that the scheduling problem of the multi-sensor is a sequential decision-making problem in an uncertain environment and with incomplete information. It is necessary to use timely information to decide the current detection scheme. The partially observable Markov decision process (POMDP) model is a theoretical tool for studying multi-stage decision-making in random environments, and provides a complete description framework for the multi-sensor scheduling problem in this paper. Posterior Cramer–Rao lower bound (PCRLB), as a measure of the optimal tracking accuracy of the system, is used to measure the detection accuracy of the sensor, so as to participate in the construction of the objective function.

The second part is the tracking algorithm, the particle filter (PF) is used to update the belief state in the model process to maintain its Markov property. At the same time, the optimization process of the beetle swarm optimization (BSO) is used to replace the resampling process of the PF algorithm to overcome the particle depletion problems of the PF algorithm, so as to improve the performance of the algorithm and the accuracy of filtering.

The third part is the solution algorithm, which uses the elephant herding optimization (EHO) to solve the solution, and by researching and improving the initialization stage, update stage and separation stage of the EHO, the performance of the algorithm is improved, and the speed and quality of the solution are improved.

## 2. Analysis of Multi-Sensor Cooperative Scheduling Model Based on POMDP

### 2.1. Framework Composition and Function

In the context of distributed defense, the multi-sensor scheduling process can be described as a Markov decision process. In a task cycle, the sensor detects the target at time k, obtains the measurement value $z_{k}$, and then formulates the sensor scheduling action for the current step according to the obtained measurement value $z_{k}$ and the corresponding constraints, so as to maximize the detection performance of the sensor and the ratio of accuracy and energy consumption, or maximize the expected return such as the minimum probability of target loss, and perform the same operation at time $z_{k+1}$ to achieve continuous and high-precision tracking of the target.

**Figure 1:**
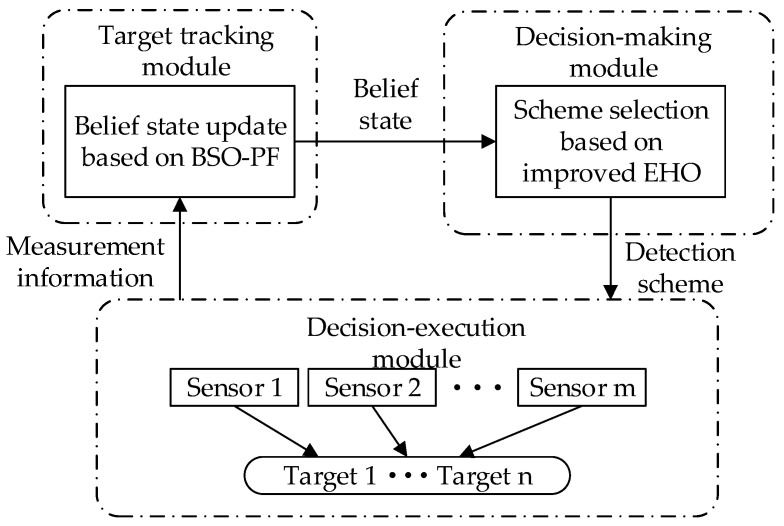
Multi-sensor collaborative scheduling framework.

The multi-sensor collaborative scheduling framework can be composed of three modules, namely the target tracking module, the plan formulation module and the plan execution module. The target tracking module is responsible for receiving the measurement information of the sensors and outputting the belief state, the scheme formulation module accepts the belief status and outputs the detection scheme, the scheme execution module receives and executes the detection scheme and outputs the measurement information. The three cooperate with each other to complete the target detection task. The multi-sensor cooperative scheduling framework is shown in Figure 1.

The target tracking module is mainly used to update the belief state, which is the source of the target belief state and one of the bases for action selection. The accuracy of target tracking is the foundation of the sensor scheduling scheme. The key point of target tracking is the prediction of the target state. However, in the actual sensor detection process, due to the performance constraints of the sensor, the high performance of the target and its complex tactical tactics, the observation of the target state is often uncertain. As a result, the state of the system is often not completely observable, and only $z_{k}$ with noise can be obtained, which cannot be directly used in the decision-making process, and it is difficult to support the subsequent attack process. Therefore, using the POMDP principle, by introducing the belief state $b_{k}$ to fully count the historical observations $z_{k}$ and actions c, and continuously updating them, the basic idea is to use the new tracking filtering algorithm to update the posterior probability distribution of the system state. It also guarantees the Markov property of the decision-making process. Therefore, the scheduling process becomes a continuous cycle of observation-belief state update-action-observation. In this paper, the PF algorithm is used to update the belief state, and the BSO algorithm is used to improve the performance of the PF algorithm and improve the tracking performance of the algorithm, which is suitable for any nonlinear system.

The program formulation module is the process of generating the sensor scheduling program. According to the detection accuracy of the target, the energy consumption of the sensor and the optimization target of the scheme, the module formulates the optimal target tracking scheme at the current moment under certain constraints. In this paper, an improved elephant herding optimization algorithm is used to solve the scheduling scheme selection problem.

The scheme execution module is mainly used by the sensor network to perform the detection task according to the established detection scheme, and obtain the measurement information of the target to support the update of the belief state and maintain the cycle of the detection process.

### 2.2. Model Elements Analysis

(1) Sensor action

Assuming that the defender deploys M sensors dispersedly according to a certain principle, at time k, there are N airborne targets. Due to the energy consumption constraints of the sensors, all sensors cannot work at the same time. Then construct the action matrix C, its elements:

(1) $$c_{ij}=\{\begin{array}{cc} 1, & Sensor\;i\;is\;added\;to\;the\;detection\;task\;for\;target\;j\\ 0, & Sensor\;i\;is\;not\;added\;to\;the\;detection\;task\;for\;target\;j \end{array},$$

(2) System state space

Define moment k, the system state is $S_{k}={\left[s_{k}^{1},s_{k}^{2},\cdots s_{k}^{N}\right]}^{T}$, and the element $s_{k}^{n}$ is denoted as $s_{k}^{n}={\left[x_{k}^{n},{\dot{x}}_{k}^{n},y_{k}^{n},{\dot{y}}_{k}^{n}\right]}^{T}$. $x_{k}^{n}$, $y_{k}^{n}$, ${\dot{x}}_{k}^{n}$, and ${\dot{y}}_{k}^{n}$ represent the vector components of the target in position and velocity.

(3) System observation set

Define time k, the system observation is the set $Z_{k}={\left[z_{k}^{1},z_{k}^{2},\cdots z_{k}^{M}\right]}^{T}$ of the measurement values of sensor m to the target, element $z_{k}^{m}$ is represented as $z_{k}^{m}={\left[z_{k}^{m,1},z_{k}^{m,2},\cdots z_{k}^{m,N}\right]}^{T}$, $z_{k}^{m,n}$ represents the measurement value of sensor m to target n at time k, $z_{k}^{m,n}={\left[\theta_{k}^{m,n},\gamma_{k}^{m,n}\right]}^{T}$, where $\theta_{k}^{m,n}$ and $\gamma_{k}^{m,n}$ represent the azimuth and oblique distance of target n relative to sensor m. The working mode of the sensor can adopt the classic AOA angle measurement mode and RSSI ranging mode.

(4) State transition law

The state transition law is determined by the state transition equation of the target. At time k, the state transition equation of target n is:

(2) $$s_{k+1}^{n}=f_{k}^{n}s_{k}^{n}+w_{k}^{n},$$

in the formula, $f_{k}^{n}$ is the state transition matrix of target n, and $w_{k}^{n}$ is Gaussian noise with the mean of 0 and the variance of $q_{k}^{n}$. Then the system state transition rate is:

(3) $$S_{k+1}=F_{k}S_{k}+W_{k},$$

in the formula, $F_{k}$ is the system transition matrix, expressed as $F_{k}=diag\left(f_{k}^{1},f_{k}^{2},\cdots ,f_{k}^{N}\right)$, $W_{k}$ is the system transition noise, expressed as $W_{k}={\left[w_{k}^{1},w_{k}^{2},\cdots ,w_{k}^{N}\right]}^{T}$, and its variance matrix is $Q_{k}=diag\left(q_{k}^{1},q_{k}^{2},\cdots ,q_{k}^{N}\right)$.

(5) Systematic observation law

The observation law of the system is determined by the measurement equation of the sensor. At time k, the measurement equation of the sensor m to the target n is:

(4) $$z_{k}^{m,n}=h_{k}^{m}\left(s_{k}^{n},c_{m,n}\right)+v_{k}^{m,n},$$

in the formula, $h_{k}^{m}$ represents the measurement equation of the sensor to the target, and $v_{k}^{m,n}$ represents the Gaussian noise with the mean of 0 and the variance of $r_{k}^{m,n}$. Then the observation law of the system is:

(5) $$Z_{k}=H_{k}\left(S_{k},C\right)+V_{k},$$

in the formula, $H_{k}$ represents the sensor measurement equation, $V_{k}$ is the observation noise, expressed as $V_{k}={\left[v_{k}^{m,1},v_{k}^{m,2},\cdots ,v_{k}^{m,n}\right]}^{T}$, and its variance matrix is $R_{k}=diag\left(r_{k}^{m,1},r_{k}^{m,2},\cdots ,r_{k}^{m,n}\right)$.

### 2.3. Optimization Goal

The purpose of sensor cooperative detection is to continuously detect the target with high precision, and at the same time, the energy consumption of the sensor network is minimized, and the selected scheme has the largest ratio of accuracy to energy consumption.

#### 2.3.1. Detection Accuracy Model Based on PCRLB

When a sensor detects the state of the target, there will be a certain error, and there will be an error every time a prediction is made. After multiple predictions, an unbiased estimator will be generated, and at the same time, along with the variance, the minimum variance will change continuously with the number of detections. The higher the detection accuracy, the smaller the variance. In contrast, the smaller the detection variance that the sensor detection can achieve, the higher the accuracy. Therefore, in order to effectively measure the detection accuracy value of the sensor, PCRLB is used as the measurement standard [22,23]. PCRLB represents the lower bound of the variance of the unbiased estimator of the system. The smaller the lower bound, the higher the detection accuracy. PCRLB usually utilizes the inverse representation of Fisher information matrix (FIM):

(6) $${\left[J_{k}\right]}^{-1}\leq E\left\{\left[{\overset{⌢}{S}}_{k}-S_{k}\right]{\left[{\overset{⌢}{S}}_{k}-S_{k}\right]}^{T}\right\},$$

in the formula, $S_{k}$ represents the real state of the target, and ${\overset{⌢}{S}}_{k}$ represents the motion state of the target. ${\left[J_{k}\right]}^{-1}$ is the PCRLB of the system, $J_{k}$ is the Fisher information matrix of the target state, expressed as:

(7) $$J_{k}=J_{s,k}+J_{z,k},$$

$J_{s,k}$ represents the a priori information FIM of the target state, which can be obtained iteratively by Equation (8).

(8) $$J_{s,k}=D_{k-1}^{22}-D_{k-1}^{21}{\left(J_{k-1}+D_{k-1}^{11}\right)}^{-1}D_{k-1}^{12},$$

in the formula, $D_{k-1}^{11}$, $D_{k-1}^{12}$, $D_{k-1}^{21}$, $D_{k-1}^{22}$ can be expressed as:

(9) $$\{\begin{array}{c} D_{k-1}^{11}=F_{k-1}^{T}Q_{k-1}^{-1}F_{k-1}\\ D_{k-1}^{12}=D_{k-1}^{21}=-F_{k-1}^{T}Q_{k-1}^{-1}\\ D_{k-1}^{22}=Q_{k-1}^{-1} \end{array},$$

Therefore, it can be obtained:

(10) $$J_{s,k}={\left(Q_{k}+F_{k}J_{k-1}^{-1}F_{k}^{T}\right)}^{-1},$$

$J_{z,k}$ represents the FIM of the measurement information, expressed as:

(11) $$J_{z,k}=\sum_{i=1}^{m}\left[{\left(H_{k}^{m}\right)}^{T}{\left(R_{k}^{m}\right)}^{-1}H_{k}^{m}\right],$$

in the formula, $H_{k}^{m}$ is the measurement array of the sensor, which represents the Jacobian matrix of $h_{k}^{m}\left(s_{k}^{n},c_{m,n}\right)$ pairs of state $s_{k}^{n}$, which is determined by the working mode of the sensor.

In summary, the PCRLB can be obtained as:

(12) $${\left[J_{k}\right]}^{-1}={\left\{{\left(Q_{k}+F_{k}J_{k-1}^{-1}F_{k}^{T}\right)}^{-1}+\sum_{i=1}^{m}\left[{\left(H_{k}^{m}\right)}^{T}{\left(R_{k}^{m}\right)}^{-1}H_{k}^{m}\right]\right\}}^{-1},$$

Using the trace of ${\left[J_{k}\right]}^{-1}$ at each moment as the accuracy index, it is expressed as:

(13) $$f_{k}=trace\left({\left[J_{k}\right]}^{-1}\right),$$

#### 2.3.2. Energy Consumption Model

Sensors will consume energy during detection. In order to maximize the continuous combat capability, the energy consumption indicator is added. Assuming that the energy consumption of the sensor scheme at time k can be expressed as:

(14) $$E_{k}=\sum_{n=1}^{N}c_{m,n}^{T}e_{m},$$

in the formula, $e_{m}$ represents the energy consumed by the sensor m to perform a detection task.

#### 2.3.3. Fitness Function

Assuming a multi-sensor multi-target background, the sensor can detect targets in one-to-one, one-to-many, and many-to-one modes, and one sensor can accurately track at most two targets at the same time.

Let $P_{n}^{k}$ denote the detection accuracy of the sensor scheme for target n at time k and be the reciprocal of $f_{k}$, expressed as:

(15) $$P_{n}^{k}=f_{k}^{-1},$$

The tracking number constraint is:

(16) $$\sum_{n=1}^{N}c_{m,n}\leq 2,$$

Then, at time k, the fitness function of the sensor scheme can be expressed as:

(17) $$\psi \left(X\right)=\frac{\sum_{n=1}^{N}P_{n}^{k}}{E_{k}},$$

Then, the optimization objective is:

(18) max[ψ(X)],

### 2.4. Task Scheduling Cycle

**Figure 2:**
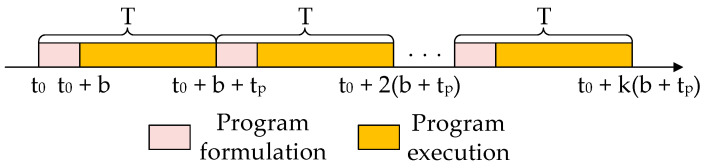
Online scheduling process.

**Figure 3:**
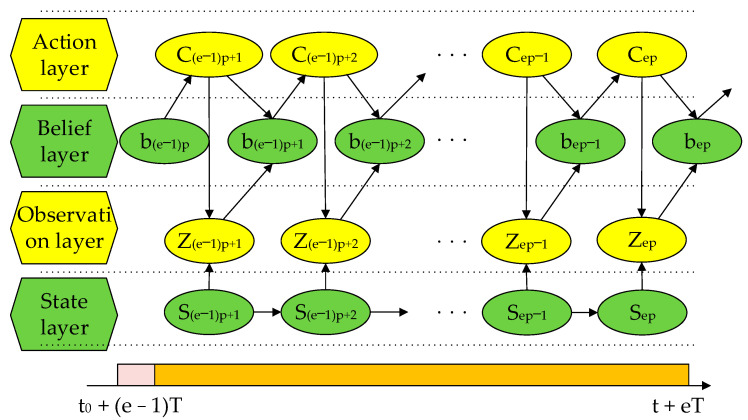
Single task cycle scheduling process.

Different from the offline scheduling process, online scheduling is a real-time continuous process. Therefore, the entire combat process is divided into e cycles T, including the program formulation and program execution stages, p sampling per cycle, the single sampling time is $t_{s}$, the total sampling time is $t_{p}=pt_{s}$, and the decision time is b, then the online scheduling process is shown in Figure 2, and the scheduling process of a single task cycle is shown in Figure 3.

## 3. A Belief State Update Method Based on Improved Particle Filter Algorithm

The particle filter algorithm [24,25] is suitable for any nonlinear system, and has higher accuracy and stability than other filtering algorithms, so this paper uses the PF algorithm to update the belief state. However, at the same time, it also has the problem of particle depletion caused by resampling. The introduction of an intelligent optimization algorithm can significantly improve this phenomenon [26,27,28]. Therefore, this paper uses the optimization process of the beetle swarm optimization algorithm to replace the particle resampling process to maintain the diversity of particles and further improve the filtering effect and stability.

### 3.1. Basic PF Algorithm

(1) Initialize the particle set

Sampling is performed according to the prior probability density function $p\left(x_{0}\right)$ (system equation) and the importance probability density function $q\left(x_{0}\right)$ (probability density function of normal distribution), and N sampled particles $\left\{x_{0}^{i},i=1,2,\cdots N\right\}$ are obtained.

(2) Calculate particle weight

The weight calculation formula is:

(19) $$w_{k}^{i}=w_{k-1}^{i}\frac{p\left(z_{k}|x_{k}^{i}\right)p\left(x_{k}^{i}|x_{k-1}^{i}\right)}{q\left(x_{k}^{i}|x_{k-1}^{i},z_{k}\right)},$$

Normalized weight:

(20) $$w_{k}^{i}=\frac{w_{k}^{i}}{\sum_{i=1}^{N}w_{k}^{i}},$$

(3) Particle resampling

Using the method of systematic resampling, the sampled particles are:

(21) $$\left\{x_{k}^{i}\right\},w_{k}^{i}=\frac{1}{N},i=1,2,\cdots ,N,$$

(4) Status output

Weighted summation of particles to get the state at the next moment:

(22) $${\overset{⌢}{x}}_{k}=\sum_{i=1}^{N}w_{k}^{i}x_{k}^{i},$$

### 3.2. BSO-PF Algorithm

The beetle swarm optimization algorithm [29] is a new intelligent optimization algorithm proposed in recent years. It has the advantages of fast calculation speed, low complexity and strong search ability in dealing with low-dimensional problems. Therefore, in the resampling stage of the PF algorithm, using the BSO performs process substitution.

Each beetle represents a particle, and the global optimization idea of the beetle swarm optimization algorithm is used to make the particle adjust its position according to the optimal information, and then change the particle distribution to move the particles to the region with high-likelihood probability, so as to achieve the purpose of optimizing the weights on the premise of maintaining the diversity of particles. The basic process of the algorithm is:

(1) Initialize the beetle swarm

Each beetle is defined as a particle, and sampling is performed according to the initialization stage of the basic particle filter algorithm.

(2) The fitness function defining the location of the beetle

(23) $$F\left(X\right)=\exp\left[-\frac{1}{2R}{\left(z_{new}\left(X\right)-z_{pre}\left(X\right)\right)}^{2}\right],$$

in the formula, R is the observation noise, $z_{new}\left(\cdot \right)$ is the observed value of the system, and $z_{pre}\left(\cdot \right)$ is the predicted value of the system.

(3) Update beetle location

It is defined that the individual extreme value of the beetle swarm is $P_{k}^{im}$, and the group extreme value is $P_{k}^{gm}$. Then the speed update formula is:

(24) $$V_{k+1}^{im}=\delta V_{k}^{im}+c_{1}r_{1}\left(P_{k}^{im}-X_{k}^{im}\right)+c_{2}r_{2}\left(P_{k}^{gm}-X_{k}^{im}\right),$$

The location update formula is:

(25) $$X_{k+1}^{im}=X_{k}^{im}+\alpha V_{k}^{im}+\left(1-\alpha \right)\varsigma_{k}^{im},$$

The position increment factor $\varsigma_{k}^{im}$ is:

(26) $$\varsigma_{k}^{im}=\delta_{k}V_{k}^{im}sign\left(F\left(X_{k}^{rs}\right)-F\left(X_{k}^{ls}\right)\right),$$

The step size $\delta_{k}$ update formula is:

(27) $$\delta_{k+1}=\varepsilon_{1}\delta_{k},$$

The search and update formula for the left and right whiskers of the beetle are:

(28) $$X_{k+1}^{ls}=X_{k}^{ls}+\frac{V_{k}^{im}d}{2},$$

(29) $$X_{k+1}^{rs}=X_{k}^{rs}+\frac{V_{k}^{im}d}{2},$$

The distance between the left and right whiskers of the beetle d is:

(30) $$d_{k+1}=\frac{\delta_{k+1}}{\varepsilon_{2}},$$

in the formula, $c_{1}$ and $c_{2}$ are learning factors, $r_{1}$ and $r_{2}$ are random numbers uniformly distributed between [0,1], α is a constant, and $\varepsilon_{1}$ and $\varepsilon_{2}$ are recursive factors, which are constants.

(4) Set the algorithm termination condition

It can be seen from Equation (23) that the fitness value of beetles is inversely proportional to the difference between $z_{new}$ and $z_{pre}$. Therefore, in order to avoid the final convergence of the algorithm, the termination fitness threshold $F_{z}$ is set. If the current function value is greater than $F_{z}$, the algorithm terminates. At this time, the particles have been gathered near the real value, so as to achieve the purpose of moving the particles to the high-likelihood probability area, and at the same time reduce the amount of calculation. If all particles are concentrated near the true value, it will reduce the diversity of particles, so that the concept of distribution density function is lost. If the function value does not reach $F_{z}$, iteratively update to the maximum number of times. After many experiments, the threshold $F_{z}$ is set to 0.73 in this paper, and the experimental results are shown in the simulation analysis section.

(5) Weight update

After the optimization of the beetle swarm optimization algorithm, the particle gradually approximates the posterior probability distribution of the particle, but the particle only relies on the difference between its own fitness value and the optimal fitness value to update the position during the optimization process, which results in the particle distribution no longer obeying $p\left(x_{k}|z_{1:k-1}\right)$, and does not conform to the basis of Bayesian filtering theory, and these particles are not suitable for direct particle filtering. So these particles are not suitable for direct particle filtering. Therefore, it is necessary to modify the weights:

(31) $$w_{k}^{i}=\frac{p\left(x_{k}=s_{k}^{i}|z_{1:k-1}\right)}{q\left(s_{k}^{i}\right)}p\left(z_{k}|s_{k}^{i}\right),$$

In this way, the particle distribution theoretically does not change the probability model, and the sampling effect is improved.

(6) Weight normalization

(32) $$w_{k}^{i}=\frac{w_{k}^{i}}{\sum_{i=1}^{N}w_{k}^{i}},$$

(7) Status output

(33) $${\overset{⌢}{X}}_{k}^{i}=\sum_{n=1}^{N}w_{k}^{n}X_{k}^{n},$$

### 3.3. Complexity Analysis

Computational complexity analysis: compared with standard PF, the BSO optimization step replaces the resampling step in BSO-PF. Suppose the number of particles is N and the maximum number of iterations is M. The position of each particle in BSO is the same, which is independent and identically distributed, and the time complexity of updating the position of each particle is 6×O(1). Therefore, for one iteration, the time complexity of the position update of all particles is 6×N×O(1), then with the maximum number of iterations M, the computational complexity of BSO-PF can be obtained as O(6×M×N), the PF resampling process involves the interactive comparison of particles, and its computational complexity is O(M×N).

Due to the setting of the threshold and the maximum number of iterations, the computational complexity of BSO-PF is at most O(6×M×N). Compared with the complexity of resampling, the optimization steps are more complicated. BSO-PF is higher than PF in operation time. By increasing the fitness threshold, the number of iterations M is moderately reduced, and the complexity is reduced. Finally, the experiment is supplemented, and the operation time of BSO-PF, basic PF, EKF and PSO-PF is compared and analyzed under the set initial conditions, which is consistent with the complexity analysis. The results are shown in Table 1.

## 4. Multi-Sensor Scheduling Scheme Solving Algorithm

In the context of distributed defense, sensors are deployed in various places according to certain principles, and have a certain scale, and the targets are often attacked in groups or in batches. Therefore, the sensor scheduling process must be fast, efficient and accurate. This requires that the scheduling algorithm can quickly and accurately obtain the sensor detection scheme at each moment. The elephant herding optimization algorithm [30,31] is a new type of algorithm developed in recent years. It has the advantages of easy implementation, high precision and fast convergence in solving resource allocation problems, and it is widely used. Therefore, this paper chooses the elephant herding optimization algorithm for strategy selection, and improves it to further improve its optimization accuracy and convergence speed.

### 4.1. Basic Elephant Herding Optimization Algorithm

In the basic elephant herding optimization algorithm, the behavior of the elephant herd is idealized into two parts, one is the clan renewal part, and the other is the clan separation part. The algorithm process is as follows:

(1) Clan update

The position of the female patriarch in the clan is the position with the highest fitness, and each elephant will be updated according to the position of the patriarch. Suppose there are n clans and each clan has j elephants, the update formula is:

(34) $$x_{new,c_{i},j}=x_{c_{i},j}+\alpha \left(x_{best,c_{i}}-x_{c_{i},j}\right)r_{1},$$

in the formula, $x_{new,c_{i},j}$ and $x_{c_{i},j}$ are the new and old positions of elephant j in clan $c_{i}$, $x_{best,c_{i}}$ is the position with the highest fitness value in the clan, α is the influence factor between [0,1], and $r_{1}$ is a random number between [0,1].

The update of the clan leader position is affected by the information of clan members, the update formula is:

(35) $$x_{new,c_{i},j}=\beta x_{center,c_{i}},$$

(36) $$x_{center,c_{i}}=\frac{1}{n_{c_{i}}}\sum_{j=1}^{n_{c_{i}}}x_{c_{i},j},$$

in the formula, β is the scale factor between [0,1], $x_{center,c_{i}}$ is the center of clan $c_{i}$, and $n_{c_{i}}$ is the number of elephants in clan $c_{i}$. It can be seen that the position of the patriarch in a clan is related to all elephants.

(2) Clan separation

During the separation process, male elephants leave the clan life alone when they become adults, which is equivalent to enhancing the global optimization ability of the algorithm. Assuming that the worst elephant in the clan is represented by a separation operator, the separation process is shown in Equation (36).

(37) $$x_{worst,c_{i}}=x_{\min}+\left(x_{\max}-x_{\min}+1\right)r_{2},$$

in the formula, $x_{worst,c_{i}}$ is the worst elephant in clan $c_{i}$, $x_{\max}$ and $x_{\min}$ are the upper and lower bounds of the space for elephants to search, and $r_{2}$ is a random number between [0,1].

### 4.2. Improved Elephant Herding Optimization Algorithm

The elephant herding optimization algorithm consists of two parts: clan update and clan separation. It has certain global optimization capabilities, but there are also some limitations.

#### 4.2.1. Limitations

(1) The initialization phase has an important influence on the rapid convergence of the algorithm and the global optimization. The basic elephant group is randomly initialized in the initialization phase, which is not conducive to the optimization process of the algorithm.

(2) In the basic elephant herd algorithm, the position of the clan head is updated only by the position information of all elephants in the clan, which lacks the global learning ability, which easily makes the clan fall into the local optimum.

(3) In the separation operation, the worst elephant $x_{worst,c_{i}}$ must leave the group. In the basic elephant herding optimization algorithm, the update method of $x_{worst,c_{i}}$ is random search, which is not conducive to giving full play to the optimization ability of the algorithm. In theory, the new position of the elephant should not be worse than the old position fitness value.

#### 4.2.2. Improved Algorithm

(1) Initialization phase

After random initialization of M elephant individuals, a reverse learning is performed, and a better elephant group is selected as the initial population. Then use the initial grouping mode of the leapfrog algorithm to divide clans [32]. Sort the fitness values, and divide the sorted elephants into N groups, and M=Np, $c_{i}$ represent the i clan, its members are $\left\{m_{i},m_{p+i},m_{2p+i},\cdots m_{\left(N-1\right)p+i}\right\}$, and the patriarch is $m_{i}$, the worst elephant is $m_{\left(N-1\right)p+i}$. In this way, the method of grouping by fitness value can make the population have higher population richness and better global search ability.

(2) Update phase

The matriarch should not only be influenced by the clan members, but also have a global vision, be able to learn from other clan chiefs, and lead the clan members to a better solution. At the same time, an adaptive influence factor β is added to adjust the effect of optimal patriarch and clan center on matriarch positions. In the early stage of the search, a larger value of β is taken to improve the global optimization ability of the algorithm. In the later stage of the search, β becomes smaller to improve the local optimization speed and quickly approach the optimal solution. The update formula is:

(38) $$x_{new,c_{i},j}=\beta x_{best,c_{i}}+\left(1-\beta \right)x_{ceenter,c_{i}},$$

(39) $$x_{center,c_{i}}=\frac{1}{n_{c_{i}}}\sum_{j=1}^{n_{c_{i}}}x_{c_{i},j},$$

in the formula, $x_{best,c_{i}}$ is the best position of all clan leaders, and β is the influence factor, which is a decreasing function of time. The formula is:

(40) $$\beta \left(t\right)=\left(\beta_{\max}-\beta_{\min}\right)\sin\left(\frac{\pi }{2}\left(1-\frac{t}{t_{\max}}\right)\right)+\beta_{\min},$$

in the formula, $\beta_{\max}$ and $\beta_{\min}$ are the maximum and minimum values of β respectively, and $t_{\max}$ is the maximum number of iterations.

(3) Separation stage

When the worst elephant in the clan arrives at the new position, compare the fitness value, if it is greater than it, replace it, if it is smaller, apply Gaussian disturbance in each dimension of the position, which not only maintains the performance of global optimization of the separation operation, but also stably improves the average fitness of the population. At the same time, the impact on the overall algorithm running speed is reduced.

Now the new position $x_{new,c_{i}}$ of the elephant is obtained according to formula (37), and then the fitness values of $x_{new,c_{i}}$ and $x_{worst,c_{i}}$ are compared. If the former is larger, it is directly replaced, if the latter is larger, a Gaussian perturbation is performed on one dimension of $x_{worst,c_{i}}$, after obtaining the new position, compare it. If the fitness value is better, replace it. Otherwise, continue to perturb. If a better fitness value is not found after repeating the maximum number of perturbations, it means that this location is not suitable for elephants to explore, and the separation operation is abandoned. The Gaussian perturbation formula for the elephant position is:

(41) $$x_{new,c_{i}}^{d}=x_{worst,c_{i}}^{d}+\left(X_{\max}-X_{\min}\right)Gaussian\left(\mu ,\sigma^{2}\right),$$

(42) d=random(1,D),

in the formula, $x_{new,c_{i}}$, $x_{worst,c_{i}}$ represent the position of the elephant before and after the disturbance, d represents any dimension of the position, D represents the total number of dimensions, $X_{\max}$ and $X_{\min}$ represent the upper and lower limits of the possible values on dimension d, respectively, μ and $\sigma^{2}$ represent the mean and variance of the Gaussian distribution, respectively.

## 5. Simulation Analysis

### 5.1. Combat Scenario

Assume that in a scenario of 40 km × 40 km, the defender deploys 100 sensors, all of which are in a standby state, regardless of the boot time, the detection task can be executed immediately after the scheme is selected. At this time, there are three attacking targets $j_{1}$, $j_{2}$ and $j_{3}$ in the air. $j_{1}$ moves in a uniform straight line at a speed of (−400 m/s, −320 m/s). $j_{2}$ makes a uniform turn, initial speed is (0, −560 m/s), turn rate is (1 degree/s), $j_{3}$ makes a maneuver turn between 41 and 50 s, turn rate is (6 degrees/s), and the rest of the time makes a uniform turn, initial speed is (−590 m/s, 0), $j_{1}$, $j_{2}$ and $j_{3}$ start at (40 km, 35 km), (35 km, 40 km) and (40 km, 31 km), respectively. Set the Q of the target as diag([0.4, 0.005, 0.4, 0.005]), the sensor ranging error $\sigma_{R}^{2}$ is 0.1^2^ km, and the sensor angle measurement error $\sigma_{\theta }^{2}$ is 0.02^2^ rad. The number of particles is 200, the simulation time is 100 s, and the sampling interval is 1 s.

### 5.2. Tracking Effect Analysis

**Figure 4:**
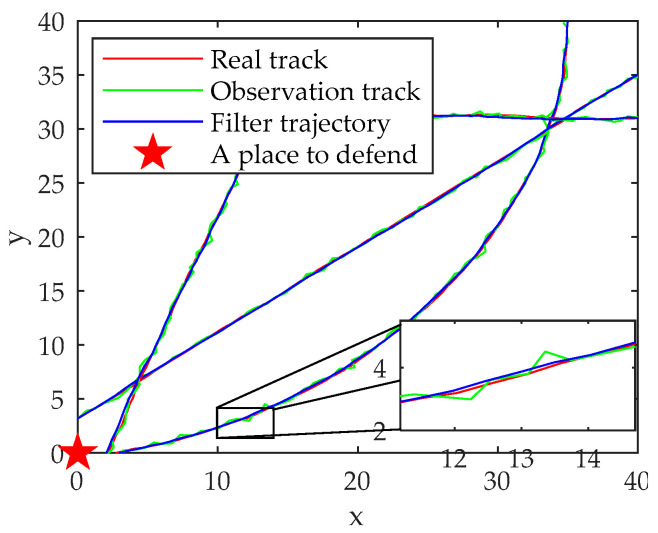
Target tracking trajectory graph.

The motion trajectories, observation trajectories and filtering trajectories of targets $j_{1}$, $j_{2}$ and $j_{3}$ are shown in Figure 4.

From the overall motion and tracking trajectory in Figure 4, the algorithm can better maneuver the target.

**Figure 5:**
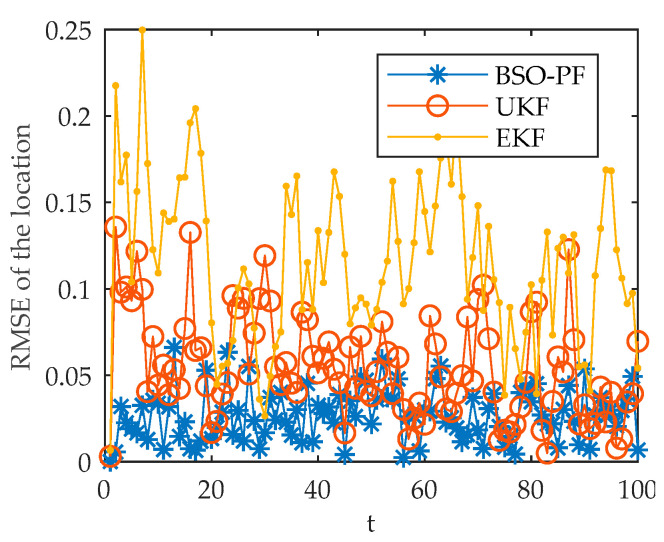
Algorithm comparison chart.

**Figure 6:**
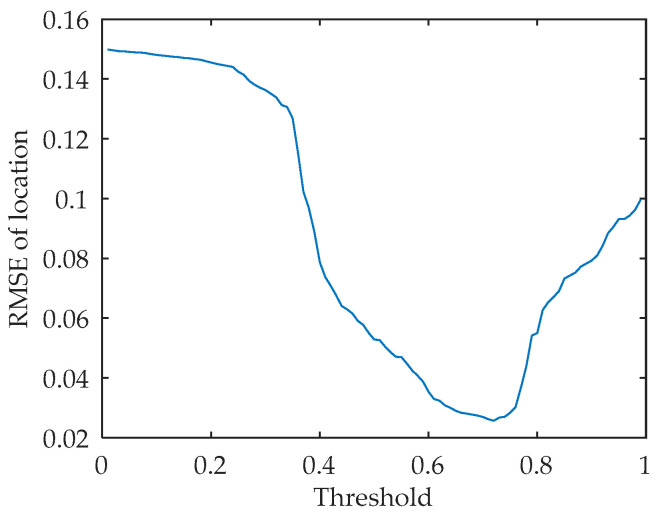
Accuracy variation at different thresholds.

Taking the position RMSE as the index and selecting the target $j_{2}$ as the simulation object, the simulation comparison and analysis of the three tracking algorithms of PF, UKF and EKF are carried out. The results are shown in Figure 5. The effect of threshold on accuracy is shown in Figure 6.

The results show that the PF algorithm has a smaller position RMSE, higher tracking accuracy and more stable tracking effect when tracking the target.

### 5.3. Analysis of Program Selection Results

#### 5.3.1. Sensor Deployment

**Figure 7:**
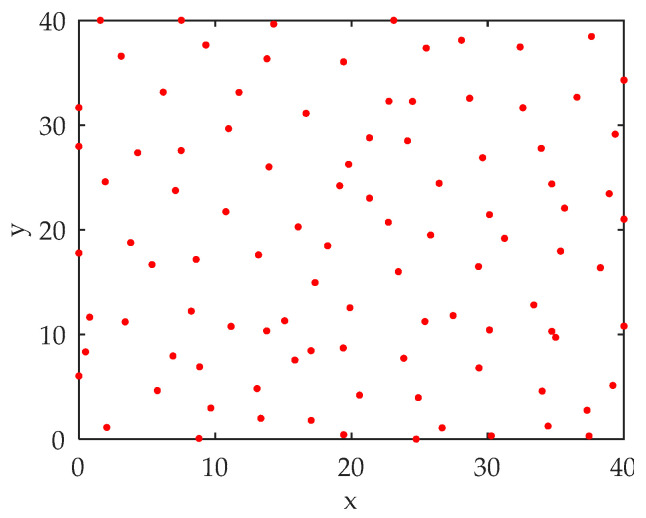
Sensor deployment plan.

Assuming that within the set range of 40 km × 40 km, the accurate detection range of the sensor is 5 km, and the method of reference [33] considers the comprehensive coverage and resource utilization for sensor deployment. The simulation result is shown in Figure 7.

In Figure 7, the red dots represent the sensor positions. At this time, the accurate detection coverage of the sensor reaches 98.63%, and the sensor resource utilization rate is the highest.

#### 5.3.2. Analysis of Multi-Sensor Scheduling Scheme

This paper compares and analyzes three scheduling methods, namely, the scheduling method, the nearest neighbor method, and the all-opening method.

Nearest neighbor method: the nearest neighbor sensor selection is performed with a range of 5 km.

All-opening method: turn on all sensors to detect and track the target.

**Figure 8:**
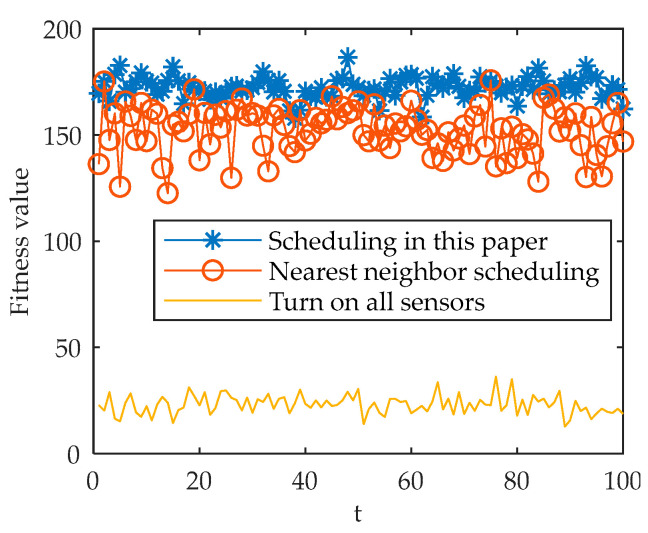
The 25 s and 80 s sensor scheme selection.

**Figure 9:**
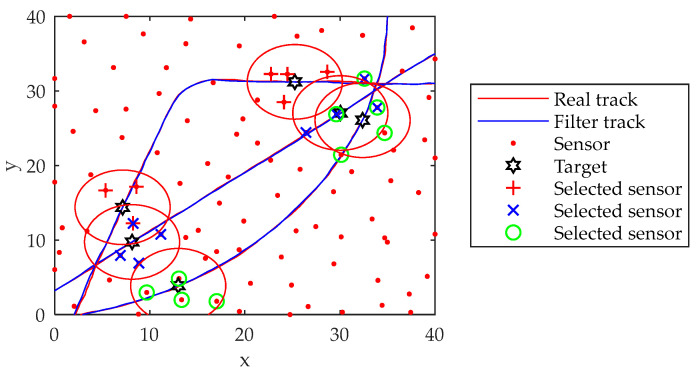
Comparison of fitness values of three methods.

The simulation results are shown in Figure 8 and Figure 9.

It can be seen from Figure 8 that the method in this paper and the nearest neighbor scheduling method select different sensors for detection at different times for the three targets. The nearest neighbor method strictly selects sensors within 5 km for detection, while the method in this paper selects a more reasonable sensor scheme according to the accuracy and energy consumption. As can be seen from Figure 9, in the simulation time of 100 s, the scheduling method in this paper has a higher fitness value than the nearest neighbor method and the all-opening method, and the sensor scheduling is more reasonable.

### 5.4. Analysis of Program Selection Results

**Figure 10:**
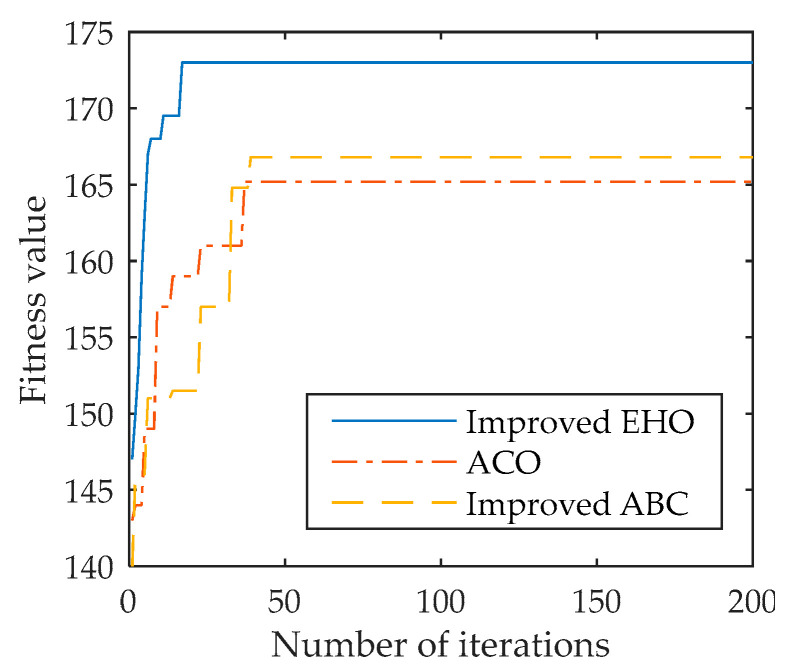
Three algorithm fitness value changes at the 25 s time.

Three algorithms, the algorithm in this paper, the ant colony optimization algorithm, and the improved artificial bee colony algorithm were selected, and the simulation and comparative analysis of the decision-making process at the time of 25 s was carried out. The results are shown in Figure 10. The whole process is simulated and compared, and the results are shown in Table 2.

Judging from the single decision at the 25 s time in Figure 10, the improved elephant herding optimization algorithm reached stability at the 16th time, and the fitness value was 173.81. Compared with the improved artificial bee colony algorithm and the ant colony optimization algorithm, it had a faster solution speed and solution accuracy. It can also be seen from Table 2 that in the 100 s simulation results, the improved elephant herding optimization algorithm had a better optimization effect than the improved artificial bee colony algorithm and the ant colony optimization algorithm, although the average fitness value was similar to the improved bee colony algorithm, it had faster convergence speed and operation time, which meets the requirements of single decision-making time.

## 6. Summarize

This paper proposes a multi-sensor cooperative scheduling model based on POMDP. Firstly, for the multi-sensor multi-objective cooperative scheduling process, a sensor cooperative scheduling framework and various model elements based on POMDP were established, so that the utilization of uncertain and incomplete target states was realized. At the same time, a PCRLB-based precision consumption ratio model was established for the optimization objective function, using PCRLB to define the detection accuracy of the sensor, it had good tracking performance and could meet the accuracy requirements to participate in the construction of the optimization objective function. In terms of algorithms, the PF tracking algorithm was used to update the belief state, and the BSO was used to improve the PF, which overcame the particle depletion problem of PF, enhanced the performance of the algorithm, and improved the accuracy of target detection. The elephant swarm optimization algorithm was used to solve the plan, and improve the basic elephant group optimization algorithm to improve the solution speed and quality of the algorithm, and further improve the speed and accuracy of the scheduling plan.

The simulation results show that the proposed model and algorithm can be well adapted to the multi-sensor multi-target scheduling problem under the background of distributed defense. The proposed improved algorithm has better performance, can adapt to the dynamically changing battlefield environment, and can achieve continuous, accurate and fast tracking of maneuvering targets, which has certain reference significance.

The next step will be to study the sensor-fire coordinated strike mechanism, model and algorithm in the context of distributed defense. Research into the firepower–target allocation model and high-performance algorithms is needed, focusing on exploring the matching of sensors and firepower units, guidance models and algorithms in complex and changeable environments, in order to achieve the best launch of the fire unit by using the sensor under the background of distributed defense for the purpose of intercepting as soon as possible and as far as possible. At the same time, based on the existing sensor scheduling model, trying to apply technologies such as 5G, 6G and the IoT to the sensor network, and explore new models and new algorithms that can improve the intelligence level, collaborative detection accuracy, and anti-interference ability of sensor networks, while reducing network energy consumption.

## Figures and Tables

**Table 1:** Operation time comparison.

Parameter	PF	EKF	PSO-PF	BSO-PF
N = 100	0.1253	0.1185	0.2025	0.1872
N = 200	0.1896	0.1809	0.2984	0.2863

**Table 2:** Comparison and analysis of performance of three algorithms.

Algorithm	Average Fitness	Average Number of Iterations	Average Operation Time
Improved elephant herding optimization algorithm	172.38	18.65	0.32
Improved bee colony algorithm	167.69	32.34	0.48
Ant colony algorithm	162.37	39.41	0.53

## Data Availability

Data are contained within the article.
